# High serotonin levels during brain development alter the structural input-output connectivity of neural networks in the rat somatosensory layer IV

**DOI:** 10.3389/fncel.2013.00088

**Published:** 2013-06-07

**Authors:** Stéphanie Miceli, Moritz Negwer, Fenneke van Eijs, Carla Kalkhoven, Ilja van Lierop, Judith Homberg, Dirk Schubert

**Affiliations:** Department of Cognitive Neuroscience, Centre for Neuroscience, Donders Institute for Brain, Cognition, and Behaviour, Radboud University Nijmegen Medical CentreNijmegen, Netherlands

**Keywords:** serotonin, SERT, barrel cortex, somatosensory, columnar circuitry, pyramidal cell, spiny stellate cell, morphology

## Abstract

Homeostatic regulation of serotonin (5-HT) concentration is critical for “normal” topographical organization and development of thalamocortical (TC) afferent circuits. Down-regulation of the serotonin transporter (SERT) and the consequent impaired reuptake of 5-HT at the synapse, results in a reduced terminal branching of developing TC afferents within the primary somatosensory cortex (S1). Despite the presence of multiple genetic models, the effect of high extracellular 5-HT levels on the structure and function of developing intracortical neural networks is far from being understood. Here, using juvenile SERT knockout (SERT^−/−^) rats we investigated, *in vitro*, the effect of increased 5-HT levels on the structural organization of (i) the TC projections of the ventroposteromedial thalamic nucleus toward S1, (ii) the general barrel-field pattern, and (iii) the electrophysiological and morphological properties of the excitatory cell population in layer IV of S1 [spiny stellate (SpSt) and pyramidal cells]. Our results confirmed previous findings that high levels of 5-HT during development lead to a reduction of the topographical precision of TCA projections toward the barrel cortex. Also, the barrel pattern was altered but not abolished in SERT^−/−^ rats. In layer IV, both excitatory SpSt and pyramidal cells showed a significantly reduced intracolumnar organization of their axonal projections. In addition, the layer IV SpSt cells gave rise to a prominent projection toward the infragranular layer Vb. Our findings point to a structural and functional reorganization of TCAs, as well as early stage intracortical microcircuitry, following the disruption of 5-HT reuptake during critical developmental periods. The increased projection pattern of the layer IV neurons suggests that the intracortical network changes are not limited to the main entry layer IV but may also affect the subsequent stages of the canonical circuits of the barrel cortex.

## Introduction

Serotonin (5-hydroxytryptamine; 5-HT) modulates key processes of mammalian brain development (Gaspar et al., 2003; Daubert and Condron, 2011; van Kleef et al., 2012). Throughout critical periods of neural development, extracellular 5-HT homeostasis is maintained by the serotonin transporter (SERT), responsible for the reuptake of 5-HT at the synapse (Blakely et al., 1991). SERT function can be modulated by the common 5-HTTLPR polymorphism in humans (Lesch et al., 1996; Champoux et al., 2002) or by a mutation of the SLC6SA4 gene in rodents (Olivier et al., 2008) which both leads to a general increase in extracellular 5-HT brain levels. Elevated 5-HT levels during critical developmental stages have been associated with changes in cognitive function, emotional processing as well as sensory perception (Canli and Lesch, 2007; Homberg et al., 2010). Previous studies in rodent models have shown that important structural changes in the neural circuitry underlie the observed behavioral phenotypes that result from a disrupted 5-HT homeostasis (Persico et al., 2001; Salichon et al., 2001; Esaki et al., 2005; Canli and Lesch, 2007; Wellman et al., 2007; Lee, 2009; Riccio et al., 2011).

Perceiving and correctly interpreting sensory information in the mature brain requires a high degree of precision in the topographical organization of the sensory pathways. Because of the strong topographic relationship of the whisker receptive fields mapped onto layer IV barrels (Woolsey and Van der Loos, 1970; Diamond, 1995), the rodent primary somatosensory cortex (S1) represents a unique structure for the study of cortical development. Following whisker deflection, the spatio-temporal information concerning the stimulus is transmitted along the lemniscal pathway via the brainstem and thalamus before reaching S1. During neural development, afferent axonal projections from neurons in the ventroposteromedial nucleus of the thalamus (VPM) grow by relying on the temporal expression of a series of guidance cues eventually resulting in a topographic innervation of the input layers of S1 (López-Bendito and Molnár, 2003; Bonnin et al., 2007). The development of primary sensory cortices is also strongly dependent on a tight regulation of extracellular 5-HT concentrations regulated by SERT, transiently expressed on growing thalamocortical afferents (TCAs) from E11 to P7 in rats (Bennett-Clarke et al., 1996; Lebrand et al., 1996, 1998; Rebsam et al., 2002; Gaspar et al., 2003). Increasing extracellular 5-HT levels, either by blocking its reuptake or its degradation, have been shown to cause a deregulation of guidance cues resulting in a reduction of axon terminals innervating S1 (Cases et al., 1996; Salichon et al., 2001; Lee, 2009). Also, presynaptic glutamatergic release from the TCAs instructs the neuronal clustering of postsynaptic excitatory cells in the typical barrel formation (Narboux-Neme et al., 2012). Serotonin (5-HT1-B) receptor is co-expressed on TCAs during development, and its activation has been shown to negatively regulate glutamatergic vesicle release at the TC synapse (Laurent et al., 2002). A reduced thalamic innervation accompanied with a decreased synaptic transmission has been shown to produce an alteration in the topography of the layer IV barrel field pattern (Cases et al., 1996; Narboux-Neme et al., 2012; van Kleef et al., 2012). The main excitatory target neurons of the TCA projections are the pyramidal and spiny stellate (SpSt) cells in layer IV, whose dendritic fields orient and cluster around the incoming thalamic input (Staiger et al., 1996; Datwani, 2002). Interestingly, Lee (2009) have demonstrated morphological changes in dendritic organization following a pharmacological increase of 5-HT. The efferent axonal projection from layer IV toward the associative supragranular layers differs greatly between both classes of excitatory cells (Staiger et al., 2004; Feldmeyer, 2012). Whereas SpSt cells are considered to be the neuron type that most distinctively reflects the columnar organization of S1, both on the structural and functional level by projecting within the home column (HC) and keeping information segregated, pyramidal cells project to neighboring columns (NCs) to allow integration of information between multiple columns (Schubert et al., 2003). Changes in the input-output microcircuitry of these layer IV neurons could considerably alter the intracortical processing of somatosensory information (Feldmeyer et al., 2013).

In order to study the effect of increased developmental 5-HT levels on both the afferent and efferent connectivity of the excitatory layer IV cell population, we used juvenile SERT knockout (SERT^−/−^) rats, known to exhibit 9-fold increases in brain 5-HT levels (Homberg et al., 2007). We first performed a classical characterization of our model by examining the posteromedial barrel subfield (PMBSF) anatomy of S1 using a cytochrome oxidase staining. We then validated the effect of high 5-HT on the anatomical organization of TCAs by analyzing the morphology of thalamic afferents arising from the VPM nucleus to the input layer IV using *in vitro* biocytin tracing. Our main goal was to quantify the structural changes in the dendritic and axonal arborizations of pyramidal and SpSt cells and to further analyze their intrinsic electrophysiological properties using the whole cell patch clamp technique. A changed organization of the thalamic afferents, combined with elevated extracellular 5-HT levels could alter the cortical microcircuitry as well as the physiological mechanisms involved in the early intracortical signal processing of somatosensory information.

## Materials and methods

### Animals

Experiments were performed on male juvenile (postnatal day 21–25) Wistar rats. SERT^−/−^(Slc6a4^1Hubr^) rats were generated by ENU-induced mutagenesis (Smits et al., 2006). All animals were bred and reared in the Central Animal Laboratory of the Radboud University Nijmegen Medical Centre (Nijmegen, The Netherlands). Breeding animals were derived from outcrossing heterozygous (SERT^+/−^) knockout rats for eight generations. Experimental animals were derived from homozygous breeding. We genotyped the animals routinely in order to confirm their genetic background. Animals were supplied with food and water *ad libitum* and were kept on a 12 h:12 h dark:light cycle (lights on at 0600 h). All experiments were approved by the Committee for Animal Experiments of the Radboud University Nijmegen Medical Centre, Nijmegen, The Netherlands, and all efforts were made to minimize animal suffering and to reduce the number of animals used.

### Morphological analysis of the barrel field

#### Cytochrome oxidase staining and analysis

Animals were anesthetized with isofluorane and decapitated. The brains were extracted and cortices were dissected and placed between two glass slides compressed within a distance of 1.2 mm in a PFA 4% solution for 48 h. The glass was removed and the tissue was postfixed overnight before being transferred to PBS 0.1 M. The tissue was embedded in 2% agarose and cut at 60 μm with a vibratome (Leica). CO activity was revealed as described by Wong-Riley and Welt (1980). In brief, sections were incubated in phosphate buffer (0.1 M; pH 7.4) containing (mg/ml): 0.6 cytochrome *C*, 0.5 diaminobenzidine, 45 sucrose and 0.3% catalase for 4 h at 40 °C.

#### Morphometric quantification of the barrel field

**Figure 1:**
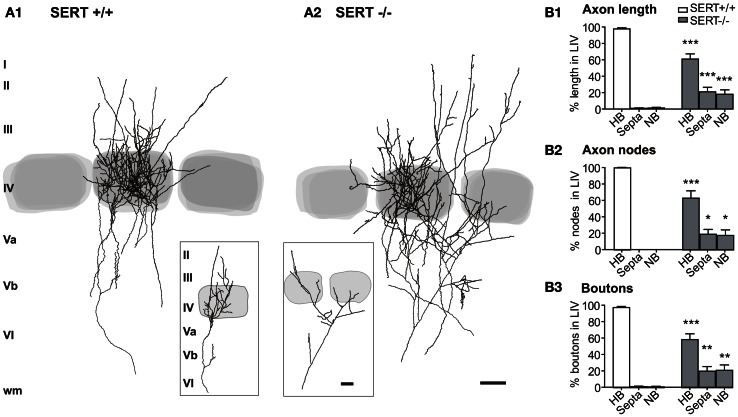
**Thalamocortical afferent projection pattern in the layer IV**. Overlay of 5 reconstructions for the SERT^+/+^ **(A1)** and SERT^−/−^ **(A2)** rats, respectively, in reference to the barrel borders stained by cytochrome oxidase. Percentages are given for the total axon length **(B1)**, number of nodes **(B2)**, and number of boutons **(B3)** innervating the HB, S, and NB (SERT^+/+^ *n* = 11; SERT^−/−^ *n* = 8). Gray shaded areas indicate the position of the respective home and adjacent barrels. Data are mean ± SEM, asterisks mark significant differences between genotypes. ^*^P < 0.05; ^**^P < 0.01; ^***^P < 0.001.

The CO stained tissue including the PMBSF barrel field was photographed at 5× magnification and pictures were analyzed using NIH ImageJ software. The external contour of barrels was outlined and areas were measured. The width of the septa between adjacent barrels was defined by the length of the line segment between two barrel borders of a straight line connecting the two barrel centroids (Figure 1).

### Electrophysiology and thalamocortical axon tracing

Acute thalamocortical (TC) slices from the rat S1 containing the pathway from the thalamus to the barrel cortex (Agmon and Connors, 1991) were used for both electrophysiology and TCA tracings. Following anesthesia and decapitation, brain tissue containing the barrel cortex was excised, quickly removed from the skull, and stored in ice-cold artificial cerebro-spinal fluid (ACSF) oxygenated with carbogen (95% O_2_, 5% CO_2_). ACSF consisted of (in mM): 124 NaCl, 1.25 NaH_2_PO_4_, 26 NaHCO_3_, 2 CaCl_2_, 5 MgCl_2_, 3 KCl, 10 glucose at pH 7.4. The hemispheres were separated and cut with a 55° angle from the midline according to the rat brain coordinates of Land and Kandler (2002). The tissue block containing the region of interest was glued to a chilled Vibratome platform (Microm HM 650 V, Microm, Germany) and slices (300 μm thickness for electrophysiology and 600 μm for TCA tracings) were cut. The slices were stored in an incubation chamber containing carbogenated ACSF at room temperature for at least 1 h, then transferred to the recording chamber and submerged in 32°C ACSF (Ca^2+^ 2 mM, Mg^2+^ 1.8 mM) at a flow rate of 1 ml/min.

#### Electrophysiology

Layer IV pyramidal cells were identified using an upright microscope (Axioskop FS, Carl Zeiss, Göttingen, Germany) fitted with 2.5× and 40× objectives. The barrel field was visualized at low magnification and the individual cells were selected within the barrels at high magnification using an infrared enhanced quarter-field illumination. Somatic whole-cell recordings were performed using borosilate glass pipettes with a tip resistance of 4–6 MΩ. Patch pipettes were filled with (in mM): 13 KCl, 117 K-gluconate, 10 K-HEPES, 2 Na_2_ATP, 0.5 NaGTP, 1 CaCl_2_, 2 MgCl_2_, 11 EGTA, and 1% biocytin. Membrane capacitance and series resistance were not compensated. Cells were selected at a minimum depth of −60 μm to retain the maximum network and minimize cutting artifacts. Each cell was characterized for its resting membrane potential and passive and active intrinsic membrane properties by injection of a series of depolarizing pulses until reaching action potential (AP) firing. Electrophysiological data was not corrected for a liquid junction potential of ca. −10 mV.

#### Signal acquisition and analysis

The signals were amplified (SEC-05L; npi-electronics, Tamm, Germany), filtered at 3 kHz, and digitized using an ITC-16 interface (Instrutech, Great Neck, NY). Data were recorded, stored, and analyzed with PC-based software (TIDA 4.1 for Windows; Heka Electronik, Lambrecht, Germany). After recording, the slices were photographed in the bath chamber to document the topography of barrel-related columns and laminae as well as the respective position of the patch electrode. The slices were then fixed in 4% buffered paraformaldehyde and stored at 4°C overnight.

### Tracing of thalamocortical axons

Acute TC brain slices (600 μm thickness) were obtained in the same manner as for the electrophysiology slice preparation. Following the vibratome slicing, brain slices were positioned on an interface chamber (Harvard Apparatus) superfused with ACSF and maintained at 32°C. A maximum of 2 acute slices per hemisphere were used in order to preserve the complete VPM to S1 axonal pathway. The VPM nucleus of the thalamus was identified under binocular magnification and a biocytin crystal (Sigma) was positioned at the center. The slices were maintained in the chamber for a period of 6 h allowing active transport of the biocytin along the afferents (King et al., 1989). The slices were then fixed overnight in a 4% PFA solution and cryoprotected in 30% sucrose solution before cryostat reslicing to 300 μm thickness. The tissue was processed for ABC-DAB staining [adapted from Staiger et al. (2004)]. In short, slices were permeated with Triton-X (0.5%), endogenous peroxidase activity was blocked by 1 h incubation with H_2_O_2_ (1%) and the slices were incubated with ABC (1:400; Vector Laboratories, Burlingame, CA) for 48 h at 4°C. Peroxidase was revealed by incubation with DAB + H_2_O_2_, and the staining was intensified by brief (1–8 min) incubation with AgNO_3_, followed by 10 min AuCl for enhanced photoresistance. The sections were counter-stained for CO histochemistry to visualize the TCA position within the barrelfield using a protocol as described in section Morphological Analysis of the Barrel Field.

### TCA and single cell *morphometric analysis*

Following the staining procedure, the brain slices containing labeled TCAs and single layer IV neurons, respectively, were analyzed using an upright brightfield microscope (Zeiss AxioImager A1) connected to the computerized reconstruction system Neurolucida (software Vers. 10, Microbrightfield Europe, Germany). For 3-dimensional reconstruction of both, TCA and single excitatory neurons in the layer IV, we used a 40× objective (Zeiss EC Plan-Neofluar, numerical aperture 0.75. The quantitative morphometrical analysis was performed by using NeuroExplorer software (software Vers. 10, Microbrightfield Europe, Germany). In order to allow layer and column specific quantitative analysis, we compartmentalized the tissue in terms of barrel/septum associated columns and the different layers under low magnification (2.5× or 5×). The borders and positions of the layer IV barrels was done based on the picture photographs of the respective acute slice preparation.

For the analysis of single neuron somatodendritic morphometric properties, we determined the largest size of the soma, the vertical and horizontal span of the dendritic field, the number of dendrites, the number of dendritic ends, the total dendritic length, the number of nodes, and the covered surface (calculated by a 2-dimensional convex hull estimation of the structure perimeter). For the axonal morphometric properties of TCAs and single neurons, we quantified the total length, the number of endings and nodes, the covered surface as well as the number and density of boutons. The boutons were identified as a distinct, point like swelling of minimum 2× the local axon thickness. The data were not corrected for the histochemistry related tissue shrinkage.

For the reconstruction of TCAs, we selected slices that contained distinguishable terminal clusters arising from a moderate amount of individual axon projections in the cortical layersVI/Vb. For single neuron reconstructions, we used the somatodendritic morphology of the biocytin stained neurons to discriminate between the two main classes of excitatory neurons in S1 layer IV, SpSt, and pyramidal neurons (Pyr). Our sample of somatodendritically reconstructed pyramidal neurons also contained a small number of star pyramidal cells which showed the typical non-skirt like organization of basal dendrites (2 out of 12 SERT^+/+^; 2 out of 15 SERT^−/−^). With no reports showing a cell class specific difference in the functional properties of these two cell types (Schubert et al., 2003), we pooled star pyramidal and pyramidal neurons. The axonally reconstructed neurons contained pyramidal neurons only. The low prevalence of star pyramidal cells in our sample can possibly be explained by a bias toward the triangular shaped somata during visual pre-selection for electrophysiological recording. The morphometric statistical analysis was carried out in SPSS (SPSS Inc., Chicago, IL).

### Statistical analysis

All data were acquired blindly and tested for normal distribution using a Shapiro–Wilk test. For the electrophysiological analyses, we used a multivariate analysis (MANOVA) to test for statistically significant differences between the two genotypes and a discriminant analysis to test for differences on the cellular population level. For the anatomical analyses, we performed a Two-Way ANOVA followed by a Bonferroni correction for *post-hoc* pairwise comparisons. All values are given as mean ± standard error of mean (SEM).

## Results

We investigated the effect of high 5-HT levels on the development of the afferent projections from the thalamus to the input layer of S1 in juvenile (P21-P25) SERT^−/−^ rats, compared to SERT^+/+^ rats with “normal” brain 5-HT levels. To this end, we first characterized the morphological properties of the terminal axonal projections of VPM neurons to the layer IV of S1. We then investigated the overall barrel field organization as well as the morphological and intrinsic functional properties of the main excitatory target neurons of VPM axons in the layer IV.

### Blocking 5-HT reuptake during development alters thalamocortical projections and organization of the barrel field in S1 cortex

#### Reduced “home” barrel specific innervation by TCAs in SERT^−/−^ rats

The typical somatotopic axon patterning in S1 consists of segregated thalamic afferents synapsing onto the layer IV cell clusters of the barrels. In the “normally” developed rat somatosensory system, single TC afferents of the VPM predominantly form their terminal clusters in one individual barrel of the cortical layer IV (Jensen and Killackey, 1987; Diamond, 1995; Bureau et al., 2006; Meyer et al., 2010; Oberlaender et al., 2012a,b) and its respective barrel associated cortical column. In SERT^−/−^ mice this topographical organization has been reported to be impaired (Persico et al., 2001). To evaluate the thalamic afferent distortion in our SERT^−/−^ rat model, we evaluated differences between the organization of terminal projections of individual TCAs of SERT^+/+^ (*n* = 11) and SERT^−/−^ rats (*n* = 8) by means of their (i) total axon length found above the level of cortical layer Vb, (ii) their level of arborization, and (iii) the number of presynaptic boutons within the layer IV. Furthermore, we quantified the lateral axonal extensions in reference to the home barrel (HB), septa and neighboring barrels.

In both SERT^+/+^ and SERT^−/−^ rats, the VPM projections revealed distinguishable terminal axon clusters in S1 layer IV which were aligned with the CO staining of the large barrels. Within S1, between layers Vb and Va, TCAs gave rise to several side collaterals and continued toward layer IV where they formed extensively arborized axonal clusters. We classified the barrel that was vertically aligned with the arising TCA in the deeper infragranular layers as the HB. Besides the rich innervation of layer IV, in both genotypes the collaterals often extended into the supragranular layers II/III.

A striking difference between SERT^+/+^ and SERT^−/−^ rats was apparent with TCAs of the SERT^−/−^ rats giving rise to fewer axon collaterals within the HB Layer IV. Furthermore, the terminal fields were more widespread laterally and less confined to the HC (Figures 1A,B). Superimposing several of the reconstructed TCAs with respect to the position of their HB still revealed a dense and distinguishable cluster of axons within the HB and a more diffuse innervation of the septa and NC. For a more detailed study of TCAs, we performed a quantitative morphometric analysis of axonal parameters (axonal length, the number of nodes, and number of endings, bouton number and density; Table 1) in reference to the relevant compartments of the barrel field (HB, septa and NB). The mean bouton densities in the present study are comparable to previous findings (Lu and Lin, 1993) and in both genotypes, the boutons were evenly distributed over the axon branches within layer IV.

**Table 1:** **Structural organization of the thalamocortical afferent innervation of the barrel cortex**.

		**Length [μm]**	**Nodes**	**Boutons**	**Bouton density (*n*/100 μm)**
Total	SERT^+/+^	2976 ± 302	28.6 ± 3.5	632 ± 74	21.2 ± 1.2
	SERT^−/−^	4903 ± 823^**^	31.6 ± 3.3	1137 ± 160^*^	24.2 ± 1.1
HC L4	SERT^+/+^	1905 ± 155	24.6 ± 3.4	452 ± 44	23.8 ± 1.2
	SERT^−/−^	1227 ± 211^***^	10.8 ± 3.3^*^	280 ± 41^**^	24.1 ± 2.2
NC L4	SERT^+/+^	25.1 ± 17.3	0.0 ± 0.0	5.1 ± 3.5	22 ± 7.8
	SERT^−/−^	354.1 ± 116.9^***^	1.8 ± 0.7^*^	93.6 ± 29.8^*^	27.1 ± 3.9
Septum	SERT^+/+^	19.2 ± 9.6	0.0 ± 0.0	7.0 ± 3.8	32.9 ± 10.7
	SERT^−/−^	415.8 ± 110.5^***^	3.0 ± 1.2	101.6 ± 31.9^*^	27.1 ± 3.9
HC L2/3	SERT^+/+^	426.7 ± 127.8	0.1 ± 0.1	108.7 ± 37	24.6 ± 1.4
	SERT^−/−^	536.8 ± 151.6	2.3 ± 0.7	144.6 ± 40.2	28 ± 1.7
NC L2/3	SERT^+/+^	33.3 ± 33.3	0.0 ± 0.0	11.8 ± 11.8	–
	SERT^−/−^	370.5 ± 181.7^**^	1.8 ± 2.2	111.8 ± 55.9	28.7 ± 1.8
Sept L2/3	SERT^+/+^	8.2 ± 8.2	0.0 ± 0.0	2.4 ± 2.4	–
	SERT^−/−^	297.8 ± 123.4^***^	1.1 ± 0.5	75.0 ± 31.0	23.7 ± 2.5

*HC, home column; NC, neighboring column. Data are means ± SEM*.

* *p < 0.05*;

** *p < 0.01*;

*** *p < 0.001*.

Axon length, nodes, and relative bouton distribution across the 3 compartments (HB, S, and NB) were significantly different for both genotypes (*p* < 0.0001; Two-Way ANOVA; Figure 1B). While in SERT^+/+^ rats between 89.9 and 100% of the TC axon was restricted to the HB (97.75 ± 1.41%), SERT^−/−^ rats showed only 60.91 ± 6.4% in the HB with an increased lateral innervation of the septa and the neighboring barrel (*p* = 0.001; Figure 1B1). In the latter, only 1 out of the 8 reconstructed TCAs remained exclusively within the barrel associated column. The remaining SERT^−/−^ TCAs had between 41 and 67% of their axon restricted to their HB. Also, while the total number of nodes and boutons per TCA were similar (Table 1), they were both found in higher percentages in their neighboring compartments compared to SERT^+/+^ TCAs (% nodes in HB: SERT^+/+^ 100 ± 0%, SERT ^−/−^ 63.2 ± 8.9%, *p* = 0.004; % boutons in the HB: SERT^+/+^ 97.7 ± 1.3%, SERT^−/−^ 58.6 ± 7.1%, *p* = 0.001). Also, the range of reconstructed projections was broader in SERT^−/−^ (2632–9346 μm) than in SERT^+/+^ (1323–4890 μm). Taken together, TCAs of SERT^−/−^ rats innervate a larger cortical surface and are less confined to their HB within S1.

#### Barrel field formation is distorted but not abolished in SERT^−/−^

**Figure 2:**
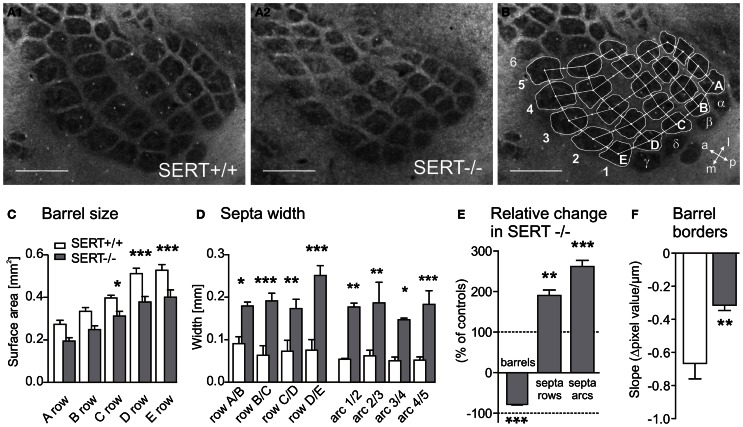
**Representative cytochrome oxidase stained tangential sections of layer IV SERT^+/+^ (A1) and SERT^−/−^ (A2) P21 rats (SERT^+/+^*n* = 6; SERT^−/−^*n* = 6)**. The sections were photographed and barrel areas (**C** and **E**) and septal distances (**D** and **E**) were quantitatively analyzed **(B)**. Barrel areas of SERT^−/−^ rats were determined corresponding to rows A-B: 1–4; C-D-E: 1–5, septa were divided into rows A–E and Arcs 1–5. The edges of the barrel borders were evaluated in the gray level pixel values (Δ GL) over distance from the barrel to the septa **(D)** and showed a reduced steepness in SERT^−/−^ compared to SERT^+/+^ **(F)**. Scale bars = 1 mm. ^*^P < 0.05; ^**^P < 0.01; ^***^P < 0.001.

Synaptic transmission of peripheral input from the thalamus to the cortex is crucial for the development and refinement of layer IV topographic maps. Previous studies have shown a reduced definition of the barrel borders or in some cases, a complete abolishment of barrel field formation in rodents having been exposed to high 5-HT levels during early developmental periods (for review see van Kleef et al., 2012). In order to evaluate the general barrel field morphology, cytochrome oxidase staining was used to visualize the neuronal densities of the PMBSF centers in tangential slices of SERT^+/+^ (*n* = 6) and SERT^−/−^ (*n* = 6) rats. In contrast to previous reports where high levels of 5-HT during development have completely impaired barrel field formation, we could identify a complete barrel field containing distinct barrels in our SERT^−/−^ rats (Figure 2A). However, compared to the barrel field of SERT^+/+^ rats, the barrel borders in SERT^−/−^ rats appeared less sharp and the septal areas more extensive. We quantified the area allocated to both, barrels and septa, respectively (Figure 2B). On average, SERT^−/−^ rats had a more than 25% reduced barrel size (25.3 ± 1.4%, *p* = 0.0075; Figure 2C). These differences in size were most prominent in the large barrels of the D and E rows. In contrast, in SERT^−/−^ rats, the width of the septa between the barrels was robustly increased almost 2-fold along the rows (190.3 ± 1.4%; *p* = 0.01; Figure 2D) and more than 2.5-fold along the arcs (261.9 ± 15%; *p* = 0.0075). The combination of decreased barrel size and increased septa width (see Figure 2E) resulted in a barrel field that, as a whole, remained similar in size for both genotypes.

In order to determine whether the wider septa and reduced barrels in SERT^−/−^ rats were attributable to a broadening of the barrel borders, we measured the “steepness” of the barrel edges by evaluating the change in CO staining intensity between the barrel and the septa over distance. We calculated the slope of change of the gray level pixel values (Δ GL) over distance, between the minimal (barrel) and maximal brightness (septum; Figure 2B). In our data sample, CO labeling within barrels and the respective septum was most reliable and homogeneous for the C1 and the C2 barrels. The slope of Δ GL across these barrel borders was significantly steeper in SERT^+/+^ (Δ GL/mm: −666.3 ± 87.5) than in SERT^−/−^ rats (Δ GL/mm: −314.4 ± 29.0; *p* = 0.008; Figure 2F), indicating less sharp borders between barrels and septa in SERT^−/−^. In summary, CO staining of S1 layer IV revealed a slightly altered, but not abolished barrel pattern in SERT^−/−^ rats, showing changes that indicate a spatially less organized neuronal clustering at the barrel borders within layer IV.

### Blocking of 5-HT reuptake affects structural, but not intrinsic functional properties of excitatory neurons in the layer IV barrels

The main excitatory target neurons of TCAs originating from the VPM are SpSt and pyramidal neurons within the layer IV barrels. We investigated how the elevated 5-HT levels, changes in TCA projections and altered barrel field organization in SERT^−/−^ rats affect the intrinsic electrophysiology properties of layer IV excitatory cells. We furthermore examined the afferent (somatodendritic) and efferent (axonal) organization of these layer IV neurons, which form a crucial backbone of the early intracortical signal processing of tactile sensory information. In TC slice preparations, we recorded from a total of 73 neurons which were classified according to their AP firing patterns as Regular Spiking (RS) or Intrinsic Bursting (IB) as well as their somatodendritic morphology as SpSt or pyramidal neurons (Pyr, including star pyramidal cells, see Materials and Methods). Following the recording, we determined the position of the neurons in respect to the layer IV barrel in the acute brain slice using bright field microscopy and confirmed with a subsequent CO staining.

#### Intrinsic electrophysiology of layer IV excitatory neurons

**Figure 3:**
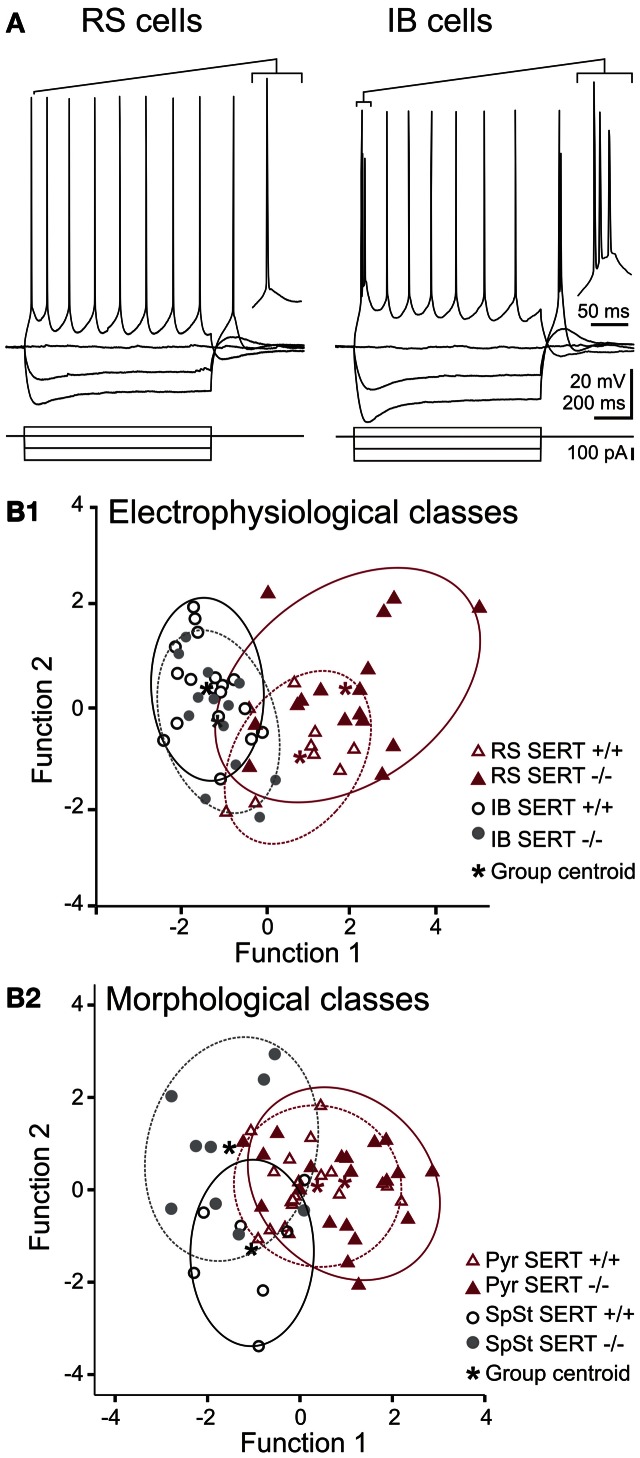
**Action potential firing pattern and discriminant analysis of electrophysiological and morphological properties of excitatory layer IV cells. (A)** Representative whole cell current clamp recordings showing regular spiking (RS) and intrinsically bursting (IB) firing patterns in SERT^−/−^ excitatory layer IV cells. Both firing patterns were observed in both genotypes (SERT^+/+^ and SERT^−/−^) as well as both morphological classes (spiny stellate cells and pyramidal cells). **(B)** Canonical score plots based on discriminant analysis of the genotype specific electrophysiological (upper panel) and morphological classes (lower panel) as *a*-priory groups. Plots were based on two functions which combined the best characteristics defining either the firing patterns (**B1**; function 1: high and low current 1st ISI; function 2: firing threshold, 2nd AP amplitude) and morphological classes (**B2**; function 1: Vrmp, high current 2nd ISI; function 2: high current 1st ISI, 2nd AP amplitude). Both analysis properties show no segregation of genotype specific populations.

We investigated whether the anatomical changes in the afferent TC pathways were accompanied by changes in excitability of the excitatory layer IV neurons. We used whole cell patch clamp recordings to investigate both passive and active intrinsic membrane properties following a sustained current injection in cells of both genotype (SERT^+/+^: *n* = 32, SERT^−/−^: *n* = 41). A summary of the most relevant intrinsic electrophysiological properties recorded is given in Table 2. In agreement with previous studies, (Chagnac-Amitai and Connors, 1989; Feldmeyer et al., 1999; Schubert et al., 2003; Staiger et al., 2004) in SERT^+/+^ rats, excitatory layer IV neurons were classified as being either regular spiking (RS; *n* = 16) or intrinsically burst spiking (IB; *n* = 16; Figure 3A). IB cells differed from RS cells most prominently in (i) eliciting an initial doublet or triplet of APs riding upon a depolarizing envelope at just suprathreshold stimulation, (ii) showing significantly shorter 1st inter-spike intervals (RS: 46.1 ± 2.1 ms; IB: 11.1 ± 4.3 ms; *p* < 0.001), and (iii) a reduced 2nd AP amplitude (RS: 69.2 ± 2.3; IB: 55.6 ± 2.3; *p* < 0.001). Across all neurons morphologically identified as SpSt cells or pyramidal cells, the two firing patterns were equally distributed (Table 2).

**Table 2:** **Electrophysiological properties of Layer IV excitatory neurons**.

	**SERT^+/+^**		**SERT^−/−^**	
	**Pyramidal cell**	**Spiny stellate cell**	**Pyramidal cell**	**Spiny stellate cell**
**PASSIVE PROPERTIES**	***n* = 22**	***n* = 10**	***n* = 30**	***n* = 11**
*V*_rmp_ [mV]	69.2 ± 1.4	−67.4 ± 1.9	−70.6 ± 1.4	−67.7 ± 2.1
*R*_m_ [MΩ]	142.5 ± 11.8	157.9 ± 23.4	128.0 ± 13.7	145.8 ± 17.6
τ_m_ [ms]	15.8 ± 1.3	15.8 ± 1.8	15.9 ± 1.2	16.1 ± 1.6
**ACTIVE PROPERTIES**				
AP threshold^1^ [mV]	−37.4 ± 1.3	−31.6 ± 1.4	−37.0 ± 1.1	−36.8 ± 2.4
AP amplitude [mV]	77.6 ± 9.9	69.5 ± 5.0	78.2 ± 9.3	76.4 ± 12.7
AP halfwidth [ms]	1.3 ± 0.4	1.4 ± 0.2	1.4 ± 0.3	1.3 ± 0.3
1st ISI – weak^1^ [ms]	27.2 ± 5.9	28.7 ± 8.4	43.6 ± 8.8	24.3 ± 8.9
1st ISI – strong^2^ [ms]	9.0 ± 0.8	7.3 ± 1.0	10.3 ± 1.1	13.7 ± 3.7
**AP FIRING PATTERN**				
Regular spiking	54.5%	40%	73.3%	36.4%
Instrinsic bursting	45.5%	60%	26.7%	63.6%

Active properties were measured by

1 just suprathreshold stimulation, eliciting 2–4 APs, or

2 by stronger depolarizing current injection, eliciting 10–14 APs. No significant differences were found between SpSt and Pyr across both genotypes. Data are means ± SEM. Pyr, pyramidal cell; SpSt, spiny stellate cell; ISI, inter-stimulus interval.

In SERT^−/−^ rats we could identify RS and IB cells (RS: *n* = 26; *IB* = 15) in both morphological classes of excitatory layer IV neurons, the only difference being that RS cells were the prevalent class amongst pyramidal cells, whereas SpSt cells tended to be more of the IB type (Table 2). Apart from this, layer IV excitatory neurons from SERT^−/−^ rats had similar membrane properties, including passive and active membrane properties (Table 1). We performed a discriminant analysis of layer IV electrophysiological properties of both SERT^+/+^ and SERT^−/−^ neurons to evaluate their relationship to either the morphological or the electrophysiological class. The results showed a clear segregation of both morphological and electrophysiological classes with both genotypes overlapping within these two groups (Figure 3B). These results indicate that the intrinsic electrophysiological properties of layer IV excitatory cells remain unchanged following exposure to high 5-HT concentrations during development.

#### Dendritic organization and axonal projections of layer IV excitatory cells

**Figure 4:**
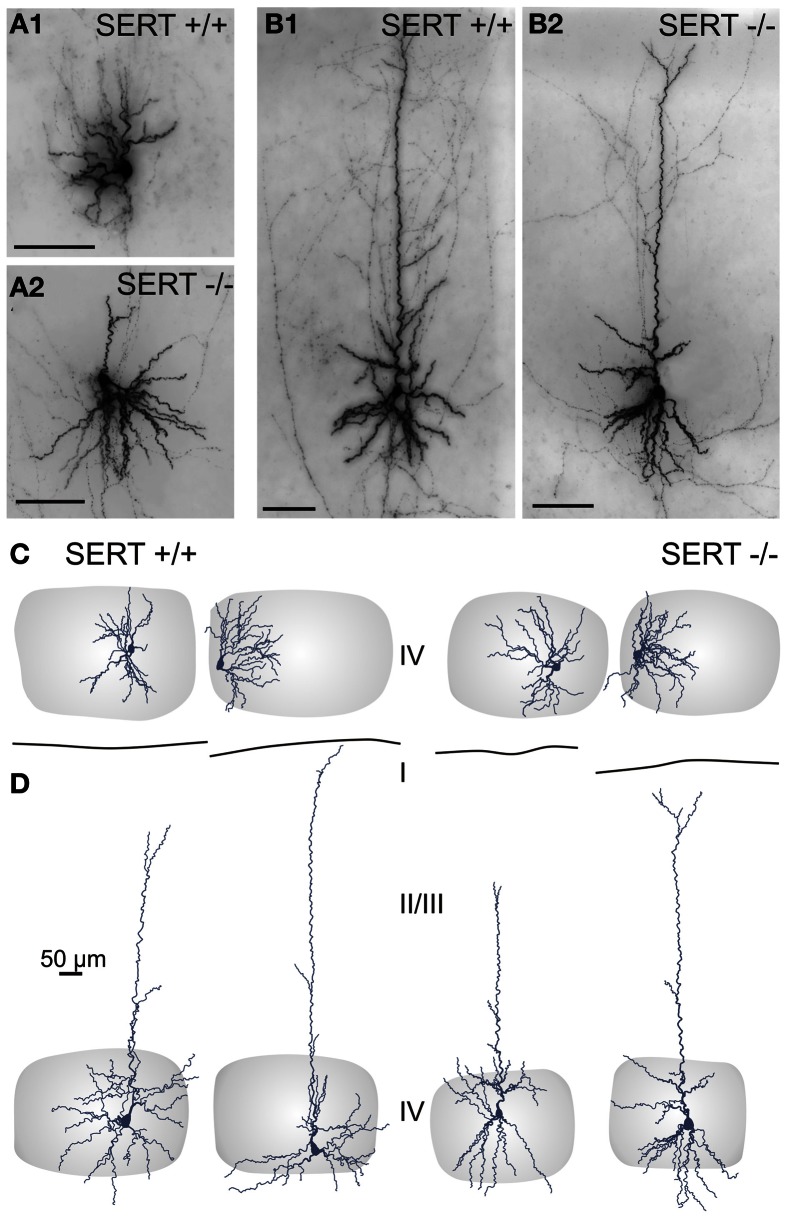
**Somatodendritic organization of excitatory spiny stellate (A,C) and pyramidal (B,D) cells in SERT^+/+^ and SERT^−/−^ layer IV barrel cortex**. Micrographs of a biocytin stained spiny stellate **(A)** and pyramidal **(B)** cells in acute slice and representative morphological reconstructions; spiny stellate **(C)** and pyramidal **(D)** of both genotypes. Gray shaded areas indicate the position of the respective home barrel.

**Figure 5:**
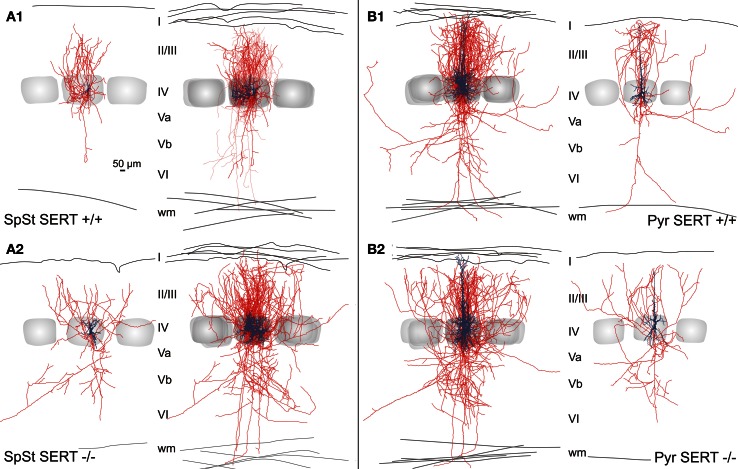
**Intracortical axonal projection pattern of excitatory cells in SERT^+/+^ and SERT^−/−^ layer IV of the barrel cortex**. Representative morphological reconstruction(s) of the somatodendritic structure (blue) and axonal projections (red) of spiny stellate cells **(A1,B1)** and pyramidal cells **(A2,B2)** in SERT^+/+^ and SERT^−/−^ cortex. Overlay of 5 reconstructed neurons aligned to their position within their home barrels which illustrates the main axonal projection patterns on the population level. Gray shaded areas indicate the position of the respective home barrel (center) and adjacent barrels.

We next investigated the detailed dendritic and axonal morphology of both classes of layer IV excitatory cells using a quantitative morphometric analysis. In the normally developed barrel cortex, layer IV excitatory neurons, and in particular SpSt cells, the dendritic and axonal organization is strongly aligned with the respective HB and its associated cortical column. We performed 3-D reconstructions of a total of 50 electrophysiologically classified and biocytin labeled neurons for somatodendritic morphological quantification (SERT^+/+^: SpSt: *n* = 10, Pyr: *n* = 12 and SERT^−/−^: SpSt: *n* = 13, Pyr: *n* = 15). Additionally we reconstructed the axon in cells where (i) the collaterals were well preserved and stained and (ii) the main descending axon remained uncut at least until it reached the deeper layer Vb of the barrel cortex (SERT^+/+^: SpSt: *n* = 7, Pyr: *n* = 6 and SERT^−/−^:SpSt: *n* = 5, Pyr: *n* = 9). Representative reconstructions and overlays of SpSt and Pyr cells are shown in Figures 4 and 5.

One key dendritic property of SpSt cells in the “normal” rodent barrel cortex is that the primary dendrites that emerge from an ovoid or round soma stay within the borders of their HB (Staiger et al., 2004). We found that the dendrites of both SERT^+/+^ and SERT^−/−^ SpSt cells rarely extended beyond a barrel border. As a consequence, neurons of both genotypes that were located close to a barrel wall gave rise to an asymmetric dendritic arborization (Figures 4A,C). This indicates that, in SERT^−/−^ rats, the somatodendritic organization is still well aligned with the general barrel pattern. Dendrites of pyramidal cells, however, typically extended into adjacent layers and into the septum, in particular the apical dendrite which in all cells of our sample reached the upper layer II/III or layer I where it terminated in a small unobtrusive tuft (Figures 4B,D).

For a more detailed quantitative evaluation of the dendritic organization in SERT^+/+^ and in SERT^−/−^ excitatory layer IV neurons, we characterized the morphometrical properties of basal and apical dendrites in terms of the total number of primary dendrites, the number of nodes, endings, dendritic span (covered surface area) and the complexity (number of endings/number primary dendrites; Table 3). Compared to SERT^+/+^, SERT^−/−^ SpSt cells showed no increase in length (SERT^+/+^: 2585.1 ± 116 μm; SERT^−/−^: 3800.3μm ± 483; *p* = 0.039) but did show an increased number of primary dendrites (SpSt: SERT^+/+^: 3.6 ± 0.2; SERT^−/−^: 5.2 ± 0.4; *p* < 0.001) and a decreased dendritic complexity (endings/ primary dendrites: SpSt: SERT^+/+^: 7.1 ± 0.6; SERT^−/−^: 4.3 ± 0.3; *p* = 0.001). SERT^−/−^ pyramidal cells also showed a significant increase in the number of branching dendrites (Pyr: SERT^+/+^: 4 ± 0.2; SERT^−/−^: 5.1 ± 0.4; *p* = 0.05) although no changes were found in their complexity (Pyr: SERT^+/+^: 3.5 ± 0.3; SERT^−/−^: 3.3 ± 0.4; *p* = 0.574).

**Table 3:** **Morphological properties of Layer IV excitatory neurons**.

	**Spiny stellate cells**		**Pyramidal cells**	
	**SERT^+/+^**	**SERT^−/−^**	**SERT^+/+^**	**SERT^−/−^**
**SOMATODENDRITIC**	***n* = 10**	***n* = 13**	***n* = 12**	***n* = 15**
Soma size (μm)	178.6 ± 18.3	151.1 ± 10.9	193.6 ± 12.9	197.5 ± 17.1
Primary dendrites (*n*)	3.6 ± 0.2	5.2 ± 0.4^**^	4.0 ± 0.2	5.1 ± 0.4^*^
Length (μm)	2427.8 ± 162	2459.2 ± 169	1802.9 ± 135	2439.8 ± 230
Nodes (*n*)	23.0 ± 1.4	21.9 ± 1.1	13.6 ± 1.1	15.8 ± 1.5
Nodes (*n*/mm)	9.6 ± 0.4	9.1 ± 0.3	7.6 ± 0.4	6.5 ± 0.3^*^
Endings (*n*)	26.7 ± 1.4	27.0 ± 1.1	17.5 ± 1.2	20.9 ± 1.7
Complexity	6.9 ± 0.8	4.6 ± 0.5^*^	3.5 ± 0.3	3.3 ± 0.4
Covered surface (μm^2^)	86225 ± 6772	70814 ± 9898	62834 ± 6626	88721 ± 8623
**APICAL DENDRITE**				
Length (μm)	1988 ± 220	1874 ± 180
Nodes (*n*)	–	–	12.6 ± 1.7	14.7 ± 2
Nodes (*n*/mm)	–	–	6.4 ± 0.4	7.7 ± 1.4
Endings (*n*)	–	–	13.8 ± 1.8	15.8 ± 2
Covered surface (mm^2^)	–	–	0.167 ± 0.029	0.142 ± 0.017
**AXONAL**	***n* = 7**	***n* = 5**	***n* = 6**	***n* = 9**
Axonal length (μm)	17201 ± 1872	18820 ± 1739	21253 ± 2046	18720 ± 1741
Axonal nodes (*n*)	79.4 ± 5.7	107 ± 4.6^**^	124.2 ± 17.7	100.7 ± 8.5
Axonal endings (*n*)	79.7 ± 5.7	108 ± 4.3^**^	123.8 ± 17.3	102.1 ± 8.6
Boutons (*n*)	3889± 447	3968 ± 335	4447± 465	4059 ± 443
Bouton density (/100μm)	21.5 ± 1.2	21.4 ± 1.3	20.9 ± 1.9	20.9 ± 0.8
Covered surface (mm^2^)	1.0 ± 0.4	1.6 ± 0.3	2.2 ± 1.0	2.2 ± 0.7

*The covered surface of a structure was calculated by a 2-dimensional convex hull estimation of the structure perimeter. Dendritic complexity is calculated by the total number of nodes/endings. Data are means ± SEM*.

* *p < 0.05*;

** p < 0.01.

The general axonal projection pattern of SERT^−/−^ SpSt and pyramidal cells showed striking differences compared to that of SERT^+/+^ cells, which was particularly obvious when superimposing reconstructed neurons in respect to their location within the HB (Bender et al., 2003; Lübke et al., 2003; Figure 5). SpSt cells in SERT^+/+^ rats revealed, individually and as a population, a projection pattern that was almost exclusively restricted to their HC. While the main descending axon projected toward the white matter with few collaterals in the deeper infragranular layers Vb and VI, recurrent collaterals formed dense projection fields within the layer IV and II/III of the HC (Figure 5A1). SERT^+/+^ pyramidal cells possessed more collaterals that projected into NCs, spanning up to 3 columns, but their main projection fields were still found within the layers IV and II/III of the HC (Figure 5B1). In contrast, in SpSt and pyramidal cells of SERT^−/−^ rats, this prominent restriction to the HC was absent. Both morphological classes gave rise to numerous collaterals that projected into the septa and NCs within layer IV and II/III (Figures 5A2, B2). Furthermore, in particular SERT^−/−^ SpSt cells possessed numerous projections into the deeper infragranular layers (Figure 5A2).

The detailed axonal properties were quantified on the basis of the axon length, number of nodes, endings, bouton number and density (for summary see Table 3). The layer and column specific properties of the axonal projections of layer IV SpSt and Pyr cells were evaluated by compartmentalized analysis, which included the HC, septal column (SC), and NC, as well as all cortical layers. Layer II/III of the home column was further subdivided into equally sized compartments reflecting the upper and lower layer II/III.

**Figure 6:**
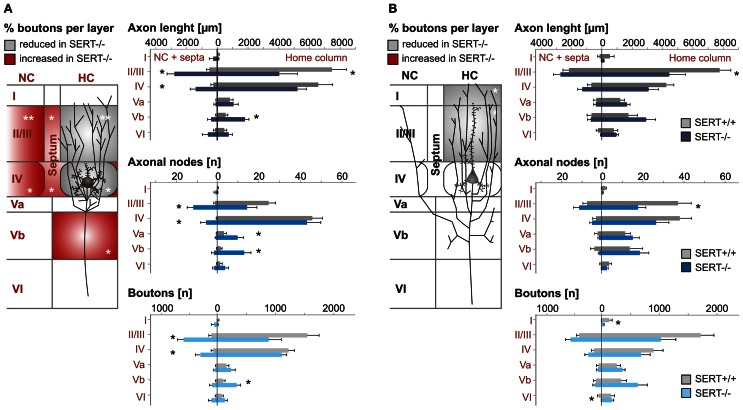
**Quantitative analysis of the axonal projection pattern of excitatory spiny stellate (A) and pyramidal cells (B) in SERT^+/+^ and SERT^−/−^ layer IV barrel cortex**. Schematic drawings (*left panels*) illustrate the significant changes in relative bouton distribution within the individual layers (I–VI), highlighting layers specific shifts from home column (HC) to septal or neighboring column (NC) and *vice versa*. Histograms (*right panels*) represent the most relevant layer specific axonal properties (length, nodes, and boutons number) per genotype. Data are mean ± SEM, asterisks mark significant differences between genotypes. ^*^P < 0.05; ^**^P < 0.01.

Our results show that while the length of the axonal projections as well as total number of boutons remained unchanged in SERT^−/−^ SpSt and pyramidal cells, the axonal pattern underwent significant cell type specific redistributions (*p* < 0.0001, ANOVA; Figures 6A,B). Whereas in SERT^+/+^ SpSt, 93% of the axon, 97% of the nodes and 94% of the boutons were found in the HC, in SERT^−/−^ rat SpSt only 68% of the axon, 79% of the nodes and 69% of the boutons were found in this compartment (*p* < 0.001, *p* = 0.002, *p* < 0.001, respectively). These redistributions of the axon projections from the HC toward septal and NCs were most prominent in the granular and supragranular layers. There, the axon collaterals established significantly more boutons, while bouton numbers in the respective layers of the HC were reduced (Figure 6A). This reduction in HC layer II/III of SERT^−/−^ rats mainly resulted from fewer axonal projections into the upper layer II/III, where axonal length and bouton numbers were halved (*p* = 0.042 and *p* = 0.048, respectively). Furthermore, we found that in SERT^−/−^ SpSt cells, all parameters of axonal projections toward the infragranular layer Vb of the HC were significantly increased by 2–3-fold, i.e., axon length (SERT^+/+^: 539 ± 149 μm; SERT^−/−^: 1805 ± 254 μm; *p* = 0.001) arborizations (number of nodes; SERT^+/+^: 1.7 ± 0.6; SERT^−/−^: 13.0 ± 1.7; *P* = 0.002) and bouton numbers (SERT^+/+^: 104 ± 32; SERT^−/−^: 333 ± 71; *p* = 0.009).

Quantitative analysis of SERT^−/−^ pyramidal cells revealed less prominent differences to those of SERT^+/+^ pyramidal cells, partially due to a higher variability in the axon projections which were extensive in both genotypes. While the qualitatively observed increase in transcolumnar projections was not significant on the quantitative level, the bouton distribution in the HC supragranular layers was significantly reduced (% layer IV boutons within HC: SERT^+/+^: 39.3 ± 5.1%; SERT^−/−^: 22.2 ± 4.0%; *P* = 0.02; Figure 6B). As in SERT^−/−^ SpSt cells, this reduction in HC associated projections was predominant for axon collaterals in the upper layers II/III (axon length: SERT^+/+^: 2801 ± 499 μm, SERT^−/−^: 4713 ± 602 μm, *p* = 0.014; number of nodes; SERT^+/+^: 19.2 ± 0.6; SERT^−/−^: 13.0 ± 1.7; *p* = 0.002) and bouton numbers (SERT^+/+^: 104 ± 32; SERT^−/−^: 333 ± 71; *p* = 0.009).

As a whole, the quantitative analysis of the morphological properties of layer IV SpSt and pyramidal cells in SERT^−/−^ rats demonstrated a cell type specific loss of home column specific restriction of axonal projections and to increased descending projections toward the infragranular layers of the S1 cortical networks.

## Discussion

There is accumulating evidence showing that 5-HT plays a key role in the network formation within the developing brain, in particular for the correct establishment of the topographic organization of the somatosensory system (Cases et al., 1996; Persico et al., 2001; Salichon et al., 2001; Gaspar et al., 2003; Xu et al., 2004; van Kleef et al., 2012). The present study shows that increased 5-HT levels during critical neurodevelopmental stages not only distorts the topographic wiring between the thalamus and the individual barrels in the layer IV of the primary S1 but also affects the efferent axonal connectivity of layer IV excitatory networks. In these networks, which form the backbone of early intracortical processing of tactile sensory information (for review see Brecht, 2007; Petersen, 2007; Feldmeyer et al., 2013), we found (i) a reduced dendritic complexity of SpSt cells within the layer IV barrels, (ii) a decrease in the intracolumnar restrictions of projections toward the associative supragranular layers II/III, and (iii) increased axonal projections toward the infra-granular layer Vb.

### Reduced topographical precision of thalamocortical innervation of the S1 cortex

During neural development, growing thalamic projections from the dorsal thalamus transiently express SERT (for review see Gaspar et al., 2003) and, guided by molecular cues that are modulated by extracellular 5-HT concentrations (Bonnin et al., 2007, 2012), reach the input layer IV of the barrel cortex around P7. Changes in 5-HT concentrations during this critical neurodevelopmental period (Erzurumlu and Kind, 2001) affects TC pathfinding and alters the barrel-like clusters of TC afferents (Bonnin et al., 2007, 2012; Li and Crair, 2011). Our analysis of individual TCAs projecting from the VPM to S1 showed that in both SERT^+/+^ and SERT^−/−^ rats, axonal clusters formed more densely in the input layer IV of S1. While normally individual thalamic axon mainly project to one barrel and its associated column (Jensen and Killackey, 1987; Diamond, 1995; Bureau et al., 2006; Meyer et al., 2010; Oberlaender et al., 2012a,b), TCAs of SERT^−/−^ rats innervated a broader field within the layer IV. These results are in agreement with previous studies in SERT^−/−^ and monoamine oxidase (MAOA)^−/−^ mice (Cases et al., 1996; Young-Davies et al., 2000; Persico et al., 2001; Salichon et al., 2001; Rebsam et al., 2002). Interestingly, we observed a clear barrel pattern in both the acute brain slices and CO-processed tissue of the SERT^−/−^ rats which allowed us to quantify the level of organization of TCA innervation in respect to the barrel associated cortical column. Our data show that in SERT^−/−^ rats, the TC innervation has lost its predominant one-to-one association to the individual HB by projecting extensively into the layer IV septa as well as into the neighboring barrels. Together with our finding that TCAs are less arborized and possess fewer boutons within their respective HB, this loss in topographic precision could result in a reduced transmission efficiency of tactile sensory signals between individual barreloids of the VPM and the cortical layer IV. *In vitro* studies have shown that the VPM to layer IV synapse forms and refines into its topographical organization during the first post-natal week, after which time the ability to induce plasticity at the layer IV synapse decreases (Feldman et al., 1999; Fox, 2002). The SERT^−/−^ phenotype observed at P21 could represent an immature system where the critical developmental time window is delayed. Recent *in vivo* studies have shown that sensory deprivation could reorganize TCA receptive fields in adult (3-month-old) animals (Oberlaender et al., 2012a,b). It remains to be investigated whether the SERT^−/−^ TCA topography can be rescued over time, through sensory experience.

### Altered but not abolished barrel field organization in SERT^−/−^ rats

A reduction in the synaptic transmission of peripheral input toward the cortex either through a reduced TCA structure or through reduced synaptic activity at the TC synapse results in an altered development of the barrel field, which could also be consequent to elevated 5-HT levels during development. Whereas some studies have reported that increases of 5-HT during critical periods of barrel cortex development, prevent the formation of the barrel field (Cases et al., 1996; Persico et al., 2001; Salichon et al., 2001; Rebsam et al., 2002), our observations point to a weaker phenotype in the SERT^−/−^ rat. Although the barrel formation is clearly visible, the delimitation between barrel and septa is more diffuse. The individual barrels of SERT^−/−^ are smaller in comparison to those of the SERT^+/+^ rats and the septal areas are significantly enlarged. Although, the CO pattern shows that the neuronal clustering of granular cells within the layer IV seems to be less defined, we found that, similar to SERT^+/+^ rats, the dendritic organization of excitatory SpSt cells of the SERT^−/−^ rats remains aligned along the edges of the barrel borders. Also, the cause and consequences of a widened septum in SERT^−/−^ rats is unknown. Interestingly, the neurons within the septa of the layer IV mainly project to the whisker related area of the primary motor cortex (M1) (Alloway et al., 2004). A modification in the circuits involved in sensorimotor control of whisking behavior could hypothetically result in changes in active sensory discrimination. Indeed, SERT^−/−^ mice have been reported to show impairments in the spontaneous gap crossing task (Pang et al., 2011).

### Intrinsic, active, and passive properties of excitatory neurons in layer IV of SERT^−/−^ rat

In the rodent somatosensory system, the main excitatory target neurons of the TCAs originating from the VPM are the SpSt and the pyramidal neurons within the barrels of S1 in layer IV (for review see Petersen, 2003; Brecht, 2007). Previous studies have shown that the layer IV excitatory neurons establish a highly interconnected local excitatory network which is capable of amplifying the sparse thalamic input before it is relayed toward the supragranular layers (Stratford et al., 1996; Feldmeyer et al., 1999, 2002; Schubert et al., 2003). Our results show that in SERT^−/−^ rats, these excitatory networks undergo cell type specific structural changes. These changes will be discussed in detail below. However, the intrinsic electrophysiological properties of layer IV excitatory neurons were not affected by excessive exposure to 5-HT during development. This key neuronal feature regulates and defines neuronal excitability as well as the temporal characteristics of the information output (Connors and Gutnick, 1990). Neither passive, nor active electrophysiological properties of both SpSt and pyramidal cells showed detectable differences across genotypes. Although *in vitro* studies have shown that acute 5-HT exposure influences neuronal excitability and synaptic plasticity (Waterhouse et al., 1986; Schmitz et al., 1995; Foehring et al., 2002) our findings imply that constant increased 5-HT levels do not lead to lasting changes in the intrinsic neuronal excitability which could play a role in modulating the reception of incoming thalamic input. Interestingly, many studies have also shown a role for 5-HT in GluR1 insertion and AMPA receptor trafficking (Derkach et al., 2007; Makino and Malinow, 2009; Jitsuki et al., 2011; Lesch and Waider, 2012). Future studies will be necessary in order to identify possible changes in plasticity and synaptic transmission within the barrel cortex of SERT^−/−^ rats.

### Cell type specific changes in general dendritic organization

The dendritic organization of neurons is regulated by presynaptic glutamatergic release as well as by surrounding monoaminergic levels (Gonzales-Burgos et al., 1996; Levitt et al., 1997; Hayashi et al., 2010). As mentioned above, SpSt cells within the barrels of layer IV typically have spatially confined dendritic trees that are restricted to the inside of a barrel and clusters around the thalamic afferent terminals (Staiger et al., 2004; Callaway and Borrell, 2011). Also, the layer IV pyramidal and star pyramidal cells, pooled in our study, give rise to an apical dendritic tree that extends the neuronal receptive surface toward the supragranular layers (Schubert et al., 2003; Staiger et al., 2004). We found no changes in the general principles of barrel-associated dendritic organization of layer IV excitatory cells of SERT^−/−^ rats. However, the SpSt and pyramidal cells both showed an increased number of elongated dendrites, that were altogether less complex in the SpSt when compared to those of SERT^+/+^ SpSt cells. In contrast, layer IV pyramidal cell dendrites were indistinguishable between genotypes. Although the loss in dendritic complexity, number of primary dendrites or elongated dendrites is in line with previous studies performed in various regions of the rodent brain (Bou-Flores et al., 2000; Norrholm and Ouimet, 2000; Lee and Lee, 2012), it contradicts others (Lee, 2009). These different results could be based on (i) the cell-type and region specific differences in 5-HT receptor expression (for review see Gaspar et al., 2003; Daubert and Condron, 2011), (ii) the age of the animals at which the study is carried out, and (iii) the timing as well as the duration of the increased exposure to the high 5-HT levels. Indeed, the effect of 5-HT receptor activation on basic dendritic (re)organization can be rapid. The reduced dendritic complexity of SpSt cells in SERT^−/−^ could lead to an altered local signal processing of tactile information [see Varga et al. (2011)]. The cell type specific nature of these dendritic alterations could be explained by the fact that the two classes of excitatory cells receive different sources of intracortical inputs, i.e., SpSt cells are almost exclusively involved in local intra-barrel signal processing, whereas pyramidal cells are wired across layers and columns (Schubert et al., 2003).

### Loss of intracolumnar dominance of excitatory layer IV output connectivity

Even though many studies have broadly investigated the link between 5-HT and the resulting disorganized topography of TC axonal projections toward S1 (for review see van Kleef et al., 2012), the resulting effect of this disorganization on intracortical connectivity has not been elucidated. In layer IV, excitatory neurons typically show two main projection domains: one within the HB in layer IV, the other in the supragranular layers II/III (Lübke et al., 2000; Petersen and Sakmann, 2000; Feldmeyer et al., 2002; Staiger et al., 2004; Lübke and Feldmeyer, 2007). There is a clear distinction between the projection patterns of layer IV SpSt and pyramidal cells. SpSt cells project almost exclusively within their home column whereas pyramidal cells, in addition to their prominent intracolumnar projections, also project into NCs (Staiger et al., 2004). Interestingly, our data demonstrate that in SERT^−/−^ rats, the excitatory cells within the layer IV have lost their prominent intracolumnar associated projection pattern, most importantly of the SpSt type. SpSt cells show a significant expansion of their projections into the septa and transcolumnar layer IV and II/III, at the expense of weakened intracolumnar projections. Also, the ascending axons of excitatory layer IV neurons preferentially form contacts with the basal dendrites of pyramidal cells within the supragranular layers II/III (Feldmeyer et al., 2002). The loss of such columnar organization within these important modules of the canonical microcircuitry of S1 (for review see Douglas and Martin, 2004; Lübke and Feldmeyer, 2007; Schubert et al., 2007) could imply important changes in the feed forward of sensory information toward the associative layers. However, the nature of the cellular population contacted by these increased transcolumnar projections still needs to be identified and could involve both excitatory and/or inhibitory neuronal populations.

Another interesting finding of this study was the presence of significantly increased downward projections of layer IV excitatory cells toward the layer Vb. The latter projections of layer IV cells toward the infragranular layers are known to be typically sparse (Gilbert and Wiesel, 1983; Callaway and Wiser, 1996; Lübke et al., 2000; Schubert et al., 2001, 2003; Staiger et al., 2004) and the consequences of these alterations may be complex since neurons in the layer Vb of S1 are a secondary target of TCAs of the VPM as well as major cortical output neurons [for review see Brecht (2007)]. Consequently, this connection may potentially modulate intracortical signal processing at the input as well as at the cortical output level. Furthermore, it has been observed that during development, layer IV excitatory cells initially project toward infra-granular layers and later on, preferentially project to supra-granular layers (Callaway and Katz, 1992; Bender et al., 2003). The extensive infra-granular projections of the SERT^−/−^ could therefore reflect an immature system.

It will be of interest to investigate whether the reorganization of intracortical wiring found in SERT^−/−^ rats is long lasting and persists throughout the life-span. Especially the intracortical connections such as the layer IV to II–III synapse which remains plastic and can be modulated throughout adulthood (Feldman, 2000; Feldmeyer et al., 2002; Fox, 2002; Bender et al., 2003; Shepherd et al., 2005) where the refinement could be compensated for at older ages.

### Functional consequences

The typical topographic columnar organization of the barrel cortex is characterized by the segregated thalamic afferents projecting to the layer IV barrels and the subsequent cell type specific intracolumnar projections toward the layers II–III. It is hypothesized that the intracortical sensory networks can correctly interpret complex spatial and temporal aspects of incoming sensory information, through the coexistence of neurons that can keep the spatial specificity of information within one column (signal segregators) and neurons that integrate information (signal integrators) across cortical columns (Schubert et al., 2003, 2007). In the barrel cortex, layer IV SpSt cells have small suprathreshold receptive fields (Brecht et al., 2004) and are thought to be the archetype of a signal segregator since they (i) receive spatially precise information via TCAs of the VPM, (ii) receive intracortical information almost exclusively from neurons in their HB, and (iii) transmit information within their column (for review, see Brecht, 2007). Our findings show that in SERT^−/−^, both TCAs as well as the intracortical projections of the excitatory layer IV cells do not possess this spatial specificity. It is of interest to evaluate the behavioral consequences of these structural deficits. Indeed, *in vivo* physiological and behavioral studies on rodents having been exposed to high 5-HT levels during brain development show changes in stimulus evoked cortical activity (Esaki et al., 2005; Pang et al., 2011) as well as a reduced tactile performance (Lee, 2009; Pang et al., 2011).

Also, it would be of great interest to clarify how the cumulated effects of the TC disorganization, the altered excitatory layer IV projections and the constant elevated 5-HT levels in SERT^−/−^ rats, could affect other crucial parts of the intracortical networks. Two of these candidate networks are (i) the associative layers II/III, central for the precise integration and interpretation of tactile sensory information arising from layer IV (Douglas and Martin, 2004; Lübke and Feldmeyer, 2007; Schubert et al., 2007), and (ii) the inhibitory interneuronal population, critical in dynamically shaping the receptive field properties and ensuring coincidence detection of excitatory signals by allowing a window of opportunity for integrating incoming thalamic information within the layer IV barrels (Gabernet et al., 2005; Sun et al., 2006; Cruikshank et al., 2007; Kimura et al., 2010).

### Conflict of interest statement

The authors declare that the research was conducted in the absence of any commercial or financial relationships that could be construed as a potential conflict of interest.
